# Seed Treatment with Sodium Nitroprusside Ensures a Long-Term Physiological and Protective Effect on Wheat under Salinity

**DOI:** 10.3390/life13071499

**Published:** 2023-07-02

**Authors:** Dilara Maslennikova, Inna Knyazeva, Oksana Vershinina, Andrey Titenkov, Oksana Lastochkina

**Affiliations:** 1 Ufa Federal Research Center, Institute of Biochemistry and Genetics, Russian Academy of Sciences, 450054 Ufa, Russia; oksana.lastochkina@ufaras.ru; 2 Federal Scientific Agroengineering Center VIM, 109428 Moscow, Russia; knyazewa.inna@yandex.ru (I.K.); vershinina.oks@yandex.ru (O.V.); tiandr1996@yandex.ru (A.T.)

**Keywords:** *Triticum aestivum* L., sodium nitroprusside, salt stress, nitric oxide, antioxidant system, photosynthetic activity, grain yield, grain amino acid profile

## Abstract

Although salinity inhibits plant growth, the use of a nitric oxide (NO) gasotransmitter can reduce its negative effects. In this study, the influence of 200 μM sodium nitroprusside (SNP) (donor of NO) on wheat plants (*Triticum aestivum* L., cv. Salavat Yulaev) in conditions of salinization (100 mM NaCl) was analyzed in pot experiments. Seed priming regulated the level of endogenous NO in normal and salinity conditions throughout the entire experiment (30 and 60 days). Salinity led to the strong accumulation of NO and H_2_O_2_, which is negative for plants, and significantly reduced leaf area and photosynthetic pigments (chlorophyll a and b and carotenoids). In addition, stress caused a drop in the content of reduced glutathione (GSH) and ascorbic acid (ASA), an accumulation of oxidized glutathione (GSSG), and significantly activated glutathione reductase (GR), ascorbate peroxidase (APX), and lipid peroxidation (LPO) in wheat leaves. SNP treatment significantly attenuated the negative effects of salinity on leaf area and photosynthetic pigments. An important indicator of reducing the damaging effect of salinity on treated plants is the stabilization of the content of GSH and ASA throughout the experiment (60 days). This condition has been associated with long-term modulation of GR and APX activity. Such an effect of 200 μM SNP may be related to its ability to reduce stress-induced accumulation of NO. Additional accumulation of proline also mitigated the negative effect of salinity on plants, and this also evidenced decreased LPO and H_2_O_2_ in them. For the first time, in natural growing conditions (small-scale field experiments), it was found that pre-sowing seed treatment with 200 μM SNP led to an improvement in the main yield indicators and an increase in the content of essential amino acids in wheat grains. Thus, SNP treatment can be used as an effective approach for prolonged protection of wheat plants under salinity and to improve grain yield and its quality.

## 1. Introduction

Wheat is one of the main cereals in the world, being a main source of calories and protein in most regions of the world. However, its production is under serious threat from a variety of abiotic factors, including salinity [1,2]. Salinity is a limiting factor increasing from arid to semiarid regions all over the world. An increase in the level of salts leads to a loss of soil productivity. Currently, approximately 1125 million hectares of lands are salt-affected, of which approximately 76 million hectares are affected by human-induced salinization [1]. Salinity affects various aspects of plants, including morphological factors, as well as several physiological and biochemical mechanisms [3,4,5,6]. Salinity causes an imbalance in the components of the antioxidant system. There is an accumulation of reactive oxygen species (ROS), a significant activation of antioxidant enzymes, and depletion of the pool of ascorbic acid (ASA) and glutathione (GSH) [5,6,7], which damages the photosynthesis and stomata state [4]. All the negative effects of the damaging effect of salinity on wheat resulted in reduced final yield production [3,4,5,6].

In recent years, studies have been focused on the use of external applications of various compounds (hormones, amino acids, minerals, etc.) to alleviate the negative impacts of salinity stress in plants [8]. For a long time, NO was considered the smallest diatomic gas (30.006 g moL^−1^), as a gasotransmitter. Today, it is positioned as a gaseous phytohormone. Even low (µM and nM) levels of NO can confer plant tolerance to a range of stresses, including salinity. NO is involved in several plant growth processes, such as germination, root/leaf development, primary and lateral root growth, respiration, photosynthesis, flowering, fruit ripening, senescence, and pollen tube growth [9,10,11,12]. As a free radical gaseous signalling molecule, NO improves plant tolerance to major stresses, such as salinity [5,6,7,9,10,11,13,14,15,16]. One of the most common ways to study NO-mediated effects on plants is through the exogenous application of chemical donors, such as sodium nitroprusside (SNP). SNP is able to reduce the adverse effects of abiotic stresses, including salinity [3,5,6,7,8,9,10,11,12,13,14,15,16]. Being an antioxidant, it can reduce the oxidative damage by detoxifying the over-produced reactive oxygen species (ROS) [6,9,14]. Exogenous SNP also improves the plant biomass production and leaf chlorophyll biosynthesis by improving the activities of antioxidants to reduce the damage caused by oxidative stress [5,6,13,14,15,16]. In addition, NO can affect the yield components and the content of amino acid in the grain [3,6,8,14,15,16]. It should be noted that there are few such works. At the same time, it is known that NO causes the accumulation of proline, an amino acid that regulates the growth and implementation of plant defense mechanisms [17,18,19].

In our previous studies, the concentration of SNP (200 μM) was chosen to have a growth-stimulating and protective effect on wheat plants [20,21]. It was found that the growth-stimulating effect is associated with the ability of 200 μM SNP to induce the accumulation of cytokinins [20] and GSH [21] in the early stages of plant ontogenesis. Pretreatment with 200 μM SNP (3-days-old seedlings incubation for 24 h) under salinity stabilized a stress-induced drop in the GSH/GSSG ratio and ASA content and resulted in additional proline accumulation. Meanwhile, it contributed to the reduction of ROS production by positively regulating the activity of SOD, APX, and GR. Particularly, an additional GR activation was found, which corresponds to the content of GSH in these plants. This was reflected in SNP-induced protective effects on the growth and leaf chlorophyll during stress and after the recovery period. Overall, SNP-pretreated plants showed the lowest MDA and electrolyte leakage under salinity, and the highest antioxidant activities, which were mainly involved in the ascorbate-glutathione cycle to maintain the balance between the ROS generation and scavenging [22]. Along with this, it was found that 200 μM SNP regulates, in normal and saline conditions, the content of salicylic acid and lignin in wheat plants. All these components play an important role in the regulation of growth and resistance of wheat plants under salinity [23]. We suggested that the chosen concentration of the SNP (200 μM) has a lasting growth-stimulating and protective effect on wheat plants. Among different modes of exogenous application, seed priming with different chemicals is an important approach in crop production [24,25,26]. Seed priming varies with the type and intensity of abiotic stresses and needs to be adopted in order to counter them. It improves the germination percentage, promotes uniformity in seedling emergence, and enhances subsequent seedling growth. At a sub-cellular level, seed priming was found to improve germination and seedling vigor by protecting cellular protein, repairing DNA damage during seed storage, improving the functioning of the protein synthesis machinery, and increasing energy levels by mitochondrial integrity. During priming, metabolic activities proceed to repair and build up nucleic acids, increase synthesis of proteins, as well as repair membranes, and enhance the activities of anti-oxidative enzymes. As a result, the germination capability and tolerance of unfavorable conditions of seeds can be promoted. Among them, activating protective antioxidant enzymes and accumulating osmoprotectants such as proline, soluble sugar, and soluble protein are the typical stress-avoidance responses [5,6,9,26,27,28]. Priming wheat seeds with the SNP is also used to study the effects of NO [5,6,9,26,27,28,29]. According to meta-analysis by Tahjib-Ul-Arif et al. [26], seed priming and foliar pretreatment were the most effective methods of NO application for plants. Moreover, one-time and regular intervals of NO treatment were more beneficial for plant growth. The optimum concentration of SNP ranges from 100 to 200 µM, and it alleviates salinity stress up to 150 mM NaCl. Furthermore, the beneficial effect of NO treatment was more pronounced as salinity stress was prolonged (>21 days) [26].

The aim of our work was to investigate how long a 200 µM SNP would be effective in providing long-term growth promotion and protection to wheat under saline conditions. We used a single soaking (priming) of seeds. Salinity was modeled with 100 mM NaCl. The physiological and biochemical parameters of wheat were studied on the 30th and 60th days. To prove the significant physiological effect of 200 μM SNP, field experiments were carried out to evaluate some components of grain yield and its amino acid profile.

## 2. Materials and Methods

### 2.1. Seed Treatment

We used wheat (*Triticum aestivum* L.) of the cv. Salavat Yulaev in the work. This wheat was obtained taking into account the peculiarities of the climate of the Republic of Bashkortostan (RB), Russia [30]. In recent years, the variety has been widely grown throughout the entire territory of the RB. Wheat seeds were obtained from Chishminsky Breeding Station, Ufa Federal Research Centre, Russian Academy of Sciences (Ufa, Russia). The seeds were sterilized in 96% ethanol for 60 s. After this, they were washed with sterile water until the smell of alcohol disappeared. A solution of SNP [SNP: (Na_2_[Fe(CN)_5_NO] 2H_2_O] was used to soak the seeds [5,6]. Pure seeds were kept for 12 h [5,6] in a solution of 200 μM SNP [20] or water (control). Seeds were treated in the dark. After soaking, seeds were dried before planting.

#### 2.1.1. Experiments on the Conditions of a Laboratory

In the first group of experiments, seeds pretreated and untreated with 200 μM SNP were grown in Petri dishes. Filter paper and water were placed in each Petri dish. Each Petri dish contained 100 seeds. At 4 days of ontogenesis, the energy of seed germination, length (roots, shoots, and the whole plant), and fresh and dry weight (FW and DW) were studied [31]. To confirm the participation of NO in SNP-induced reactions on wheat plants, we used a scavenger of NO-100 µM cPTIO(2-(4-carboxyphenyl)-4,4,5,5-tetramethylimidazoline-1-oxyl-3-oxide) [23].

#### 2.1.2. Pot Experiments under Controlled Conditions

SNP-pretreated and untreated wheat plants that were 4 days old were grown in plastic pots with soil. Plastic pots were filled with 6 kg of soil «Universal» (“Alliance”, Berezovsky, Russia) (with optimal NPK ratio, pH 6.2–6.5, moisture 65–68%). To maintain the required salinity level, the soil was carefully prepared. The soil was thoroughly crushed and dried. The soil was then saturated with 100 mM [32]. The salt soil had an ECe of 9.8 dS m^−1^. Some of the plants were planted in soil saturated with water (normal growth conditions), and the other plants were planted in soil saturated with 100 mM NaCl (stressed growth conditions). Each pot was planted with 10 plants (4 replicates for each group of experimental plants). Assessment of physiological and biochemical parameters was carried out in wheat plants on the 30th and 60th days of ontogenesis. In the course of this experiment, the leaf area and the contents of proline, photosynthetic pigments, hydrogen peroxide (H_2_O_2_), and MDA were evaluated. Additionally, the content of ascorbic acids (ASA), reduced glutathione (GSH) and its oxidized form (GSSG), and the activity of key enzymes GR and APX were assessed.

#### 2.1.3. Field Experiments

Small-scale field experiments were performed in 2021–2022 on the territory of the rural settlement “Alkinsky village council” (Chishminsky district, RB, Russia) (54°34′ N, 55°22′ E, at 116 m above sea level). The experimental field’s soil characteristics were leached chernozem (pH 5.5), humus content—8.4%, Hg—5.50 mg-EQ/100 g soil, pH_KCl_—5.9, exchangeable K—30 mg/100 g of soil, mobile P—23 mg/100 g of soil. The seeds SNP-pretreated and untreated were sown at a depth of 4–5 cm (distance between the plants–3 cm, between rows–8 cm). There were two plots (1 m^2^) for each group of experimental plants. Crop yields were measured in 2021 and 2022. The climate characteristics were as follows: the sum of rainfall in May–August was about 80 mm (2021) and 172 mm (2022). The average air temperature in May, June, July, and August was respectively 17.8, 21, 21, and 22.8 °C (2021); and 10.9, 16.7, 21, and 21 (2022). The highest temperature in 2021 was 38 °C, and in 2022, it was 24.8 °C; the lowest temperature in 2021 was 2 °C, and in 2022, it was 6 °C. Yield component parameters and grain analysis were carried out on the 90–92 days of ontogeny, according to the characteristics of the variety [30].

### 2.2. Estimation of Leaf Surface Area

Leaf area was measured by according to the Quarry and Jones equation, where leaf area = length × width × 0.75 [33]. We considered that the leaf length is at the junction of the leaf blade and the petiole. The distance between two points on the edge of the leaf blade, which is perpendicular to the axis of the length of the leaf, was considered as the width of the leaves [34].

### 2.3. Yield Components

At harvest, the shoot (above-ground part of the plant) length (cm), spike length (cm), and 1000 grains weight (g) were measured [35].

### 2.4. Grain Quality Analysis (Amino Acids, Carbohydrates, and Raw Fat)

The mass fractions of amino acids were determined through decomposition of samples by acid and alkaline hydrolysis with the conversion of amino acids into free forms, obtaining phenylisothiocarbamil derivatives. Their further separation and quantitative determination were carried out by capillary electrophoresis using a device called “Kapel-205” (Russia) in accordance with GOST 55569-2013 (National Standard of the Russian Federation) for plant materials.

The mass fraction of carbohydrates was determined using preliminary prepared sample with subsequent separation, identification, and determination of components by capillary electrophoresis. Briefly, a crushed and homogenized sample was centrifuged for 5 min at 5000 rpm and then analyzed using a capillary electrophoresis device, “Kapel-205” (Lumex-Marketing LLC, St. Petersburg, Russia), according to the certified method for measuring the mass fraction of mono- and disaccharides, and regulated in the document M 04-92-2020 “Food products, food raw materials, feed and food additives.”

The mass fraction of raw fat was determined through extracting the fat with petroleum ether in the Soxhlet apparatus. Then, the solvent was removed, and the defeated residue was weighed according to GOST ISC 13496.15-2016 (National Standard of the Russian Federation).

### 2.5. Pigment Analysis

To determine chlorophyll a and b and carotenoids content (mg g^−1^ FW), fresh leaf samples were ground in ethanol (C_2_H_5_OH) with the addition of calcium carbonate (CaCO_3_). After that, it was filtered. The resulting filtrate was analyzed accordingly [36,37].

### 2.6. Oxidative Stress Markers

The hydrogen peroxide (H_2_O_2_) production was measured according to [38] and expressed as µmoL g^−1^ FW. The lipid peroxidation was measured by measuring the malondialdehyde (MDA) content in wheat leaves as per the method of [38] and expressed as nmoL g^−1^ FW.

### 2.7. Estimation of Nonenzymatic Antioxidants

Levels of glutathione and glutathione disulfide were determined using o-phthalaldehyde (79760, Sigma-Aldrich, Burlington, MA, USA) as a fluorescent reagent, and derivatization of GSH to prevent GSH auto-oxidation was performed using *N*-ethylmaleimide (E1271, Sigma-Aldrich, Burlington, MA, USA) [38,39]. The GSH/GSSG ratio, indicating the plant redox status, was measured as total glutathione (GSH + GSSG)/oxidized glutathione (GSSG) [38]. ASA content measurements were performed as previously reported by Maslennikova et al. [38]. Determination of free proline content was according to [40] with modification [41].

### 2.8. Antioxidant Enzyme Activities

The activity of the antioxidant enzymes GR (EC 1.6.4.2) and APX (EC 1.11.1.11) was evaluated in the leaves of wheat plants. GR and APX activity were analyzed accordingly [38].

The GR and APX activities and content of GSH and GSSG were normalized to the total content of soluble proteins measured by the Bradford method (1976) [42].

### 2.9. Endogenous NO Measurement

The content of endogenous NO was determined using the Griess reagent. Nitrite and nitrate-derived NO content was measured at 540 nm using a Smart Spec Plus spectrophotometer (Bio-Rad, Hercules, CA, USA). NO content was calculated by comparison to a standard curve of NaNO_2_ and expressed as nM g^−1^ FW [43].

## 3. Statistical Analysis

Field experiments were carried out two times. The crop structure was analyzed in three biological replicates. The content of amino acids, fat, and carbohydrates was analyzed in two biological and three chemical repeats. Other experiments were repeated three times with a different number of biological repetitions, from three to five. Experimental data were expressed as means ± SE, which were calculated in all treatments using MS Excel. The significance of differences was assessed by ANOVA followed by Duncan’s test (*p* ≤ 0.05) with STATISTICA 10.0 software.

## 4. Results

### 4.1. Laboratory Experiment

**Figure 1:**
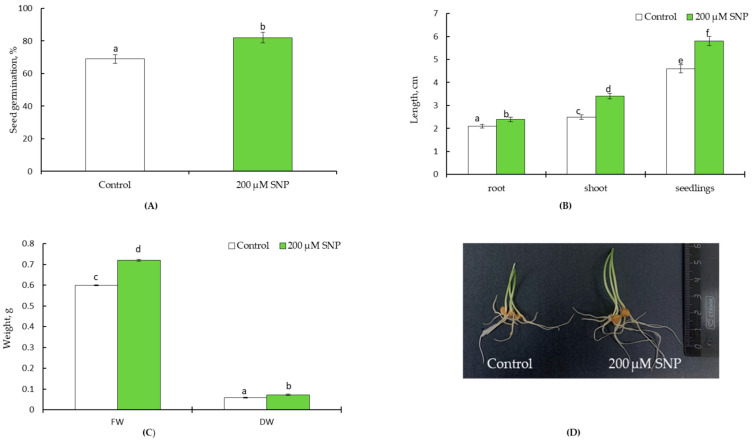
Effect of SNP pretreatment on seed germination (**A**), length (**B**), fresh weight (FW) and dry weight (**C**), and appearance (**D**) of four-day-old seedlings. All statistical differences were submitted regarding the no-treatment variant. The data are averages from three SE replications (n = 100); the averages with different letters are significantly different (*p* ≤ 0.05).

An analysis of the germination energy of treated and untreated SNP seeds showed that priming increased the germination energy of seeds by 123% (Figure 1A). Along with this, an elongation of wheat plants by 1.3 times is observed (Figure 1B). SNP pretreatment resulted in an almost 1.2-fold increase in the fresh (FW) and dry weight (DW) of plants (Figure 1C). Plants pretreated with SNP visually looked physiologically stronger compared to the control (Figure 1D).

**Figure 2:**
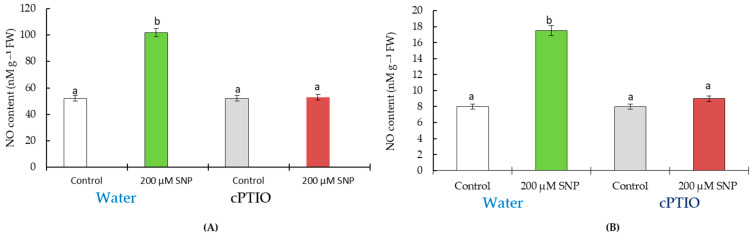
The level of NO in roots (**A**) and shoots (**B**) of four-day-old wheat seedlings. SNP treated and untreated (control) seeds were grown for four days on filter paper moistened with water (water) or scavenger of NO (cPTIO). All statistical differences were submitted regarding the no-treatment variant. The data are averages from three SE replications (n = 30); the averages with different letters are significantly different (*p* ≤ 0.05).

Data on the content of endogenous NO in wheat plants grown from untreated and SNP-treated seeds is shown in Figure 2. SNP treatment led to an increase in the level of NO in the roots by 195% (Figure 2A) and in the shoots by 210% (Figure 2B) relative to the control. The presence of cPTIO completely eliminated the SNP-induced accumulation of NO and did not affect the content of NO in control wheat plants (Figure 2).

### 4.2. Pot Experiments

**Figure 3:**
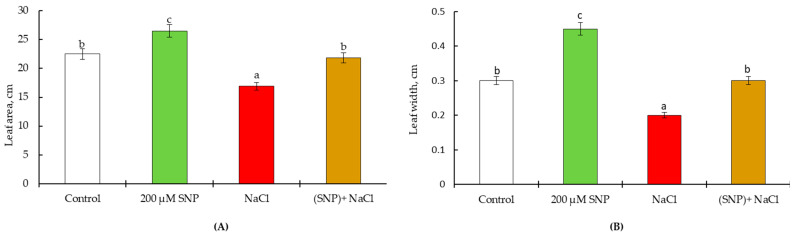
Effect of salt stress and pretreatment with 200 µM SNP on leaf area (**A**) and leaf width (**B**) in leaves of wheat seedlings. Time of stress exposure–30 days. All statistical differences were submitted regarding the control variant. The data are averages from three SE replications (n = 30); the averages with different letters are significantly different (*p* ≤ 0.05).

On the 30th day of the experiment, 100 mM NaCl led to decrease of leaf area (by 1.3 times). The treatment of plants with a donor of NO (SNP) increased the leaf area by 1.12 times under normal conditions; while under stress conditions, the leaf area of these plants remained at the control level (Figure 3A). It was revealed that a significant contribution to the leaf area is made by the width of the leaf plate. Measurements have shown that SNP treatment leads to a rise in this indicator by 150% compared to the control. Salinity decreased the leaf width, while pretreatment with SNP maintained the leaf width at the level of the control value under the same condition (Figure 3B).

**Figure 4:**
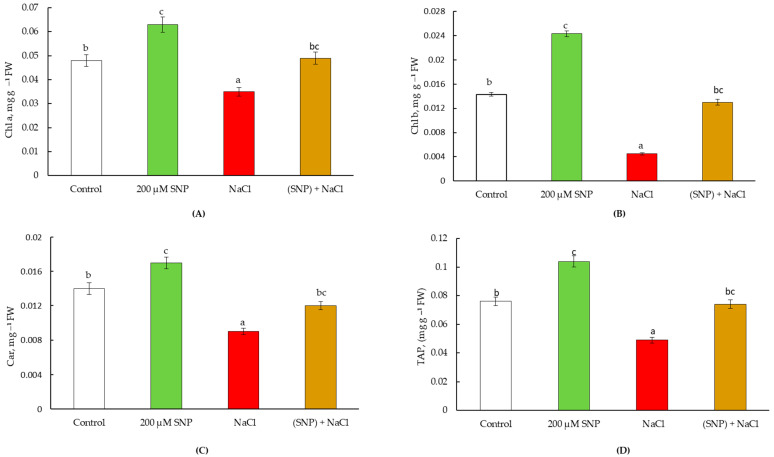
Effect of salt stress and pretreatment with 200 µM SNP on the content of chlorophyll a (**A**), chlorophyll b (**B**), carotenoids (**C**), and total amount of photosynthetic pigments (**D**) in leaves of wheat seedlings. Time of stress exposure–30 days. All statistical differences were submitted regarding the control variant. The data are averages from three SE replications; the averages with different letters are significantly different (*p* ≤ 0.05).

Our results showed that NO influences the content of photosynthetic pigments in wheat leaves under normal and salinity conditions. Seed pretreatment with 200 μM SNP resulted in an increase in the content of chlorophyll a by 30%, chlorophyll b by 60%, and carotenoids by 20% in leaves under normal growth conditions (Figure 4A–C). In these plants, the TAP was higher than the control values by 30% (Figure 4D). 

Stress led to a decrease in the content of chlorophyll a by 30%, chlorophyll b by 300%, and carotenoids by 50% relative to the control, which led to drop in TAP by 50% (Figure 4). However, pretreatment with SNP contributed to the maintenance of photosynthetic pigments at the level of the control (non-stressed) plants’ values (Figure 4A–C). This led to the stabilization of the TAP in SNP-pretreated and stressed plants (Figure 4D).

Salt stress resulted in a significant depletion (almost two times) of GSH and ASA, which was accompanied by a two-fold accumulation of GSSG and H_2_O_2_, leading to a significant increase in MDA concentration and about a five-fold drop in the GSH/GSSG ratio. Along with this, stress resulted in up to two-fold APX and GR activation, as well as proline accumulation (Table 1).

Under normal conditions, pretreatment with NO did not affect the content of GSSG, ASA, and MDA. At the same time, seed soaking with NO increased the content of GSH, GSH/GSSG ratio, proline, and H_2_O_2_. The activity of GR and APX was also higher than the control in these plants (Table 1). Under salinity in plants pretreated with NO, the accumulation of GSH was lower by 10%, GSH/GSSG ratio by 20%, and the GSSG was comparable with the control level. This change in GSH content was accompanied by additional GR activation (up to 20% above the stress level) in these plants. SNP pretreatment led to additional accumulation of proline under salinity (up to 15% above the stress level). ASA was lower (by 30%) and the activity of APX was higher (up to 38%) than the level of control values. Along with this in these plants, the content of H_2_O_2_ and MDA was up to 40% and 29% higher, respectively, than the control (Table 1).

**Figure 5:**
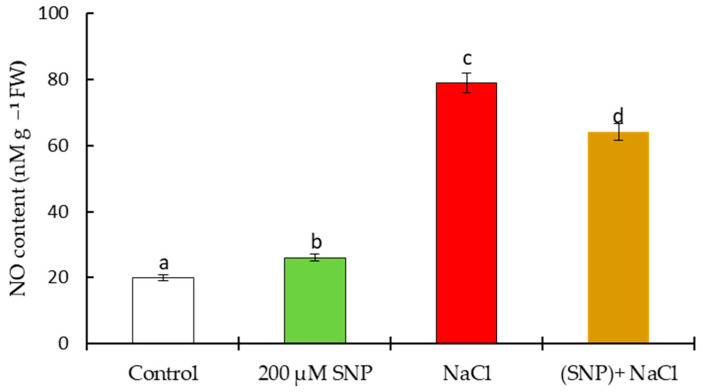
Effect of salt stress and pretreatment with 200 µM SNP on the content of NO in leaves of wheat seedlings. Time of stress exposure–30 days. All statistical differences were submitted regarding the control variant. The data are averages from three SE replications (n = 30); the averages with different letters are significantly different (*p* ≤ 0.05).

Seed treatment with 200 μM SNP under normal conditions increased the content of endogenous NO by 60% relative to the control. Salinity increased the NO content by 395% relative to the control level. In the plants pretreated with SNP under stressed conditions, the content of NO was 320% higher than the control level (Figure 5).

**Figure 6:**
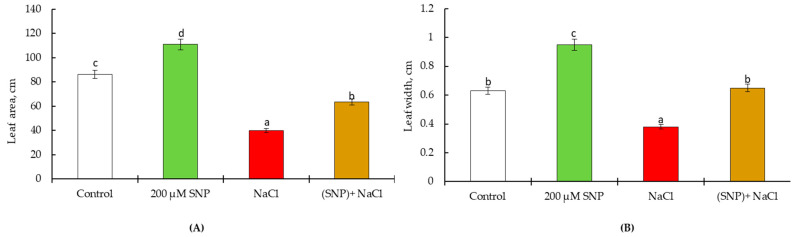
Effect of salt stress and pretreatment with 200 µM SNP on leaf area (**A**) and leaf width (**B**) in leaves of wheat seedlings. Time of stress exposure–60 days. All statistical differences were submitted regarding the control variant. The data are averages from three SE replications (n = 30); the averages with different letters are significantly different (*p* ≤ 0.05).

On the 60th day of vegetation, it was revealed that 100 mM NaCl had an inhibitory but not lethal effect on plant growth. Therefore, 100 mM NaCl leads to a decrease in leaf area by 2.2 times (Figure 6A). Treatment of plants with SNP increased the leaf area up to 1.3 times, while under NaCl stress, the leaf area of these plants was 73% lower than the control level. Under normal growth conditions, SNP pretreatment leads to an increase of leaf width by 150% relative to the control. Salinity decreased leaf width, but SNP treatment maintained the leaf width at the level of control non-stressed plants (Figure 6B).

**Figure 7:**
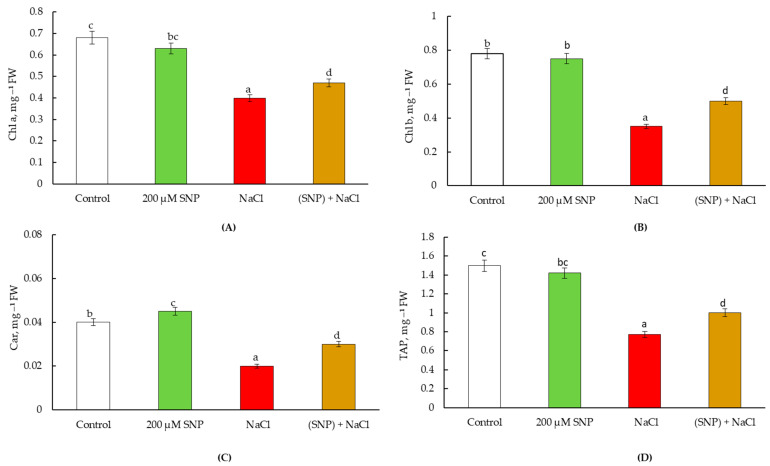
Effect of salt stress and pretreatment 200 µM SNP on content chlorophyll a (**A**), chlorophyll b (**B**), carotenoids (**C**), and the total amount of photosynthetic pigments (**D**) in leaves of wheat seedlings. Time of stress–60 days. All statistical differences were submitted regarding the control variant. The data are averages from three SE replications; the averages with different letters are significantly different (*p* ≤ 0.05).

Under normal growing conditions, it was found that on the 60th day of ontogeny, the content of chl a was 110% lower and the content of Car was 120% higher than the control level in the leaves of SNP-pretreated plants (Figure 7A,C). Contents of chl b and TAP remained on the control values (Figure 7B,D).

Salinity reduced the content of Chl a by 70%, Chl b by 220%, and Car by 200% (Figure 7A–C). This led to a 200% decrease of TAP (Figure 5D). Under stressed conditions, in SNP-pretreated plants, the content of photosynthetic pigments maintained at the 64–69% level of the control values (Figure 7A,B,D). This led to the stabilization of TAP (Figure 7D). 

Salinity causes a depletion of almost three times of GSH and ASA, which is accompanied by a 1.5-fold accumulation of GSSG and H_2_O_2_, leading to two-fold increased MDA concentration and five-fold drop in GSH/GSSG ratio. Along with this, stress led to a 1.5-fold APX and GR activation, as well as to a two-fold proline accumulation (Table 2).

Treated seeds with a donor of NO (SNP) did not affect the content GSSG, ASA, and MDA under normal conditions. At the same time, SNP treatment increased insignificantly, but reliably, the content of GSH, GSH/GSSG ratio, proline, and H_2_O_2_, as well as the activity of GR and APX (Table 2). Under salinity, SNP increased the content of GSH (by 66%) and ASA (by 33%) and decreased the ratio of GSH/GSSG by 66% relative to the control levels. GSSG content was at the control level. GR activity and proline content in these plants was slightly above the stress level. APX and H_2_O_2_ were higher, up to 20% and 38%, respectively, in comparison with control (Table 2).

**Figure 8:**
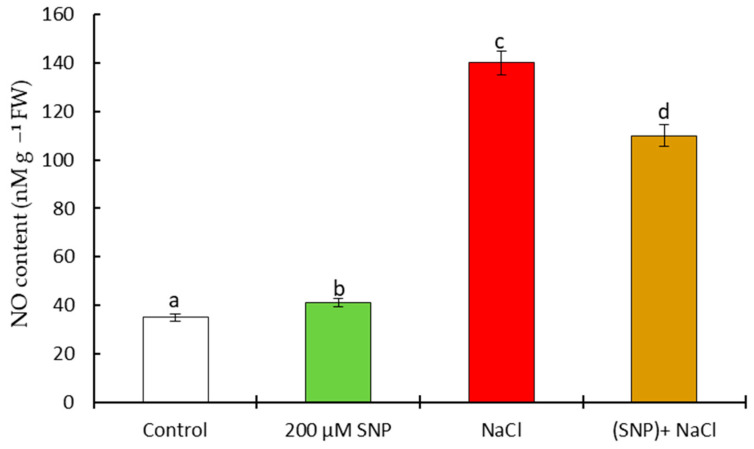
Effect of salt stress and pretreatment with 200 µM SNP on the content of NO in leaves of wheat seedlings. Time of stress exposure–60 days. All statistical differences were submitted regarding the control variant. The data are averages from three SE replications (n = 30); the averages with different letters are significantly different (*p* ≤ 0.05).

On the 60th day of vegetation in the leaves of SNP-pretreated plants under normal conditions, the content of NO was higher (by 117%) than the control. Salinity led to an increase in NO by 400%, and in the leaves of pretreated SNPs plants under stress, it was 310% higher than the control (Figure 8).

### 4.3. Small-Scale Field Experiments

Table 3 presents the results from which it can be clearly seen that pretreatment seeds of SNP increased the length of shoots (above-ground part of plants) by 1.1 times, the length of spikes by 1.25 times, and the weight of grains by 120% relative to control values.

Data on the effect of 200 µm SNP on the composition of individual amino acids, carbohydrate content (mg 100^−1^ dry weight), and crude fat (%) of wheat grain can be found in Table 4. SNP pretreatment influenced the amino acid composition of the grains, increasing the content of almost all the studied amino acids except aspartic acid + asparagine, glutamic acid, and cysteine. The content of non-essential amino acids also increased, especially the content of arginine, proline, and alanine.

SNP treatments improved the content of all essential AA in the wheat grain and increased the relation of essential AA/non-essential AA without affecting significantly the contents of carbohydrates (fructose, glucose, sucrose) and raw fat (Table 4).

## 5. Discussion

During the first stage of the study, it was of fundamental importance to evaluate the effects of seed soaking in 200 µM SNP on growth wheat parameters. Treatment with SNP to enhance seed germination was evidenced by the literature data [5,6,9,23,26,29] and the data presented in Figure 1A. Data on plant growth (Figure 1B,C) and appearance (Figure 1D) also indicate that this method of seed treatment with a nitric oxide donor has a significant growth-stimulating effect. The ability to increase the level of endogenous NO in wheat plants contributes to the implementation of the growth-stimulating effect upon 200 μM SNP treatment. The fact that it was the pre-treatment of seeds that caused the accumulation of NO is confirmed by the use of cPTIO (Figure 2). This is quite expected, since it is known that even a 3-h exposure to SNP vapours significantly promoted embryo germination of *Sorbus pohuashanensis* (Hance) Hedl. The presence of cPTIO in the germination medium of SNP-treated seeds dramatically reduced the embryo germination percentage and radicle length for sorbus plants [29]. Thus, priming wheat seeds with 200 µM SNP for 12 h had a NO-mediated growth-stimulating effect (Figure 1 and Figure 2).

These plants treated and untreated with SNP were planted in pots in the presence and absence of salinity. The experiments carried out showed a long-lasting physiological effect of 200 µM SNP on wheat plants. The findings showed that SNP (a donor of NO) stimulates the growth of wheat, as evidenced by the leaf area data. An important contribution to this process is made by an increase in the width of the leaf plate of wheat plants (Figure 2 and Figure 4). Such a stimulating effect of NO is confirmed by the literature data, as the treatment of wheat plants with 200 μM SNP led to an increase in the leaf area in seven-day-old wheat plants [44], and soaking wheat seeds with 1 mM SNP resulted in an increase in shoot mass in 50-day old plants [6,9]. The spraying of wheat plants with 5 mM SNP resulted in an increase in the area of the flag leaf and content of photosynthetic pigments [3]. Soaking wheat seeds in solutions of SNP (0.1 and 0.2 mM) for 12 h led to an increase in wheat yield and a decrease in the negative effect of sodium chloride salinity [5,6]. Thus, treatment with 200 μM SNP reduces the negative impact of salinity on the content of photosynthetic pigments (Figure 4 and Figure 7). It is known that an increase in the content of photosynthetic pigments lead to an increase in plant biomass and productivity [45,46,47]. This is supported by the data in Table 3. An important contribution to the regulation of plant growth is made by the process of cell division, mitosis. Glutathione (GSH) plays a key role in the regulation of this process [21,48]. The synthesis and accumulation of proline are associated with the organs in which cell division occurs, the apical meristems [17]. Thus, the accumulation of proline in plants under normal growing conditions also plays an important role in the growth-stimulating effect of the NO donor. During 60 days of ontogeny, an increased level of GSH is observed in SNP-pretreated plants (Table 1 and Table 2). NO and H_2_O_2_ positively regulate the rate of photosynthesis under normal conditions [6,9,11,26,49]. In addition, the content of NO remained higher than the control until the 60th day of vegetation, which confirms that all the detected effects are mediated by SNP-induced accumulation of NO. Therefore, maintaining the H_2_O_2_ and NO content above the control level in SNP-treated plants (Table 1 and Table 2, Figure 5 and Figure 8) also makes an important contribution to increasing leaf area (Figure 3 and Figure 6) and overall yield (Table 3).

Wheat is a cereal crop and a staple food for 2.5 billion people. A grain of wheat contains proteins, fats, fiber, vitamins, and trace elements [50]. A huge number of factors (temperature, humidity, fertilization, etc.) have a decisive influence on the quality of wheat grain, in particular on the content of amino acids [51,52]. In general, the physiological state of wheat plants during the growing season determines the amino acid composition of seeds. It is known that lysine, methionine, and threonine are the main limiting amino acids in wheat grain. Moreover, there are reports of their absence in the grain [53]. Our results showed that SNP leads to an additional accumulation of all essential amino acids in wheat grains of the Salavat Yulaev variety. This indicates the ability of 200 μM SNP to regulate the physiological state of wheat plants during vegetation, which leads to an improvement in the amino acid composition of grains (Table 4). Ragaey et al. [3] found that the spraying of wheat plants with SNP led to an increase in the content of essential amino acids except tryptophan. This makes the use of these grains more attractive for bakery production in order to replenish those that are indispensable in the diet of humans and animals. Thus, pretreatment with 200 μM SNP has a long-term growth-stimulating effect and improves the physiological state of wheat plants, which is reflected in an increase in grain value and yield.

Along with the physiological effect, a prolonged protective action of the donor of NO on wheat under salinity was revealed. Salinity caused a significant generation of H_2_O_2_ (Table 1 and Table 2) and NO (Figure 5 and Figure 8) throughout the experiment, which indicates the development of oxidative and nitrosative stress, which leads to damage to membrane structures and pigments [3,13,54]. This leads to slower growth and development of the leaves (Figure 2 and Figure 4). Salinity causes significant depletion of GSH and ASA, drops the ratio of GSH/GSSG, and inhibits wheat leaf growth [5,7,55]. It was shown that the treatment of *Olea europaea* L. plants with ASA and GSH significantly reduced leaf damage caused by salinity [56]. The donor of NO positively regulated plant growth through the maintenance of the level of GSH and ASA and the stabilization of the ratio of GSH/GSSG during the entire experiment. GR is a key enzyme in the maintenance of GSH under stresses [57]. Our results showed that pretreatment with SNP was able to regulate GR activity throughout the experiment. Stress caused significant GR activation, but SNP-pretreatment contributes to an additional increase of its activity (Table 3 and Table 4). APX regulates the conversion of H_2_O_2_ into H_2_O and O_2_, using an AsA as a donor [58]. Thus, the APX controls the accumulation of H_2_O_2_ in plant tissues [59]. Under stressed conditions, SNP pretreatment reduced stress-induced activation of APX, which led to the normalization of H_2_O_2_ and ASA content, too (Table 1 and Table 2). At the same time, SNP pretreatment reduced the stress-induced accumulation of NO, which indicates a prolonged SNP-induced participation in the growth-stimulating effect of 200 μM SNP (Figure 3 and Figure 6); possibly, this makes a significant contribution to the increase in wheat yield (Table 3).

The amino acid proline plays a key role in the life and development of plant resistance. In addition, proline has antioxidant properties and is involved in osmoprotection [17,60]. Salt stress exposure during 24 h induced an increase in the concentration of proline in all plant organs. In saline conditions, the application of proline improves growth by seed germination, biomass, photosynthesis, gas exchange, and grain yield in crop plants [19]. Proline increases plant resistance to salinity. We believe that the additional accumulation of proline to 60-day ontogenesis plays an important role in the SNP of induced resistance to 100 mM NaCl (Table 1 and Table 2). This ability of NO leads to a decrease in the damaging effect of salinity on seedlings (Table 1 and Table 2). NO is able to modulate the resistance of cabbage plants to 100 mM NaCl by regulating their content of proline [61]. NO and proline increase wheat’s resistance to heat stress. This effect of SNP is due to the fact that nitric oxide and proline are able to reduce the negative effects of stress by regulating redox metabolism, the accumulation of osmolytes, and photosynthetic pigments [62]. These data indicate a reduction in salinity-induced oxidative stress in SNP-pretreated plants, as evidenced by MDA values (Table 1 and Table 2). Thus, under saline conditions, pretreatment seed with 200 μM SNP had a prolonged protective effect on the growth and antioxidant system of wheat plants under salinity.

## 6. Conclusions

Based on the data obtained, it can be assumed that the revealed ability of 200 µM SNP to regulate the endogenous content of NO under normal and salinity conditions makes an important contribution to the implementation of the prolonged growth-stimulating and protective effect of 200 µM SNP on wheat. This effect is due to the fact that NO regulates the state of the antioxidant system and the content of photosynthetic pigments. Along with this, pre-sowing seed treatment with SNP positively modulates the yield and amino acid composition of grain in field-grown plants. Such data could be the basis for using NO to increase wheat grain yield and its quality.

## Figures and Tables

**Table 1:** Influence of 200 µM SNP on the content of GSH, GSSG (μmoL mg^−1^protein), GSH/GSSG ratio, ASA (mg% FW), MDA (nM g^−1^ FW), H_2_O_2_ proline (µmoL g^−1^ FW), and the state of antioxidant enzymes GR (nmoL min^−1^ mg^−1^ protein) and APX (µmoL ascorbate oxidized mg^−1^ min^−1^ protein) in leaves of wheat seedlings under salinity stress (1 mM NaCl). Time of stress exposure–30 days.

Treatment	GSH	GSSG	GSH/ GSSG	ASA	GR	APX	H_2_O_2_	Proline	MDA
Control	59 ± 2.5 ^a^	2.5 ± 0.11 ^a^	23.6 ± 0.9 ^a^	1.9 ± 0.08 ^a^	3.59 ± 0.14 ^a^	5.45 ± 0.22 ^a^	3.5 ± 0.13 ^a^	13 ± 0.52 ^a^	5.5 ± 0.22 ^a^
200 µM SNP	65 ± 2.5 ^b^	2.3 ± 0.09 ^a^	28 ± 1.1 ^b^	2 ± 0.08 ^a^	4.9 ± 0.19 ^b^	5.78 ± 0.23 ^b^	3.9 ± 0.14 ^b^	18 ± 0.75 ^b^	5.56 ± 0.22 ^a^
NaCl	25 ± 1.1 ^c^	5.8 ± 0.23 ^b^	4.1 ± 0.16 ^c^	0.6 ± 0.02 ^b^	7.8 ± 0.32 ^c^	11.08 ± 0.44	6.1 ± 0.24 ^c^	30 ± 1.2 ^c^	7.8 ± 0.32 ^b^
(SNP) + NaCl	54 ± 2.16 ^ab^	2.8 ± 0.11 ^ac^	19.2 ± 0.8 ^d^	1.4 ± 0.06 ^c^	9.2 ± 0.36 ^d^	7.58 ± 0.3 ^d^	4.8 ± 0.19 ^d^	34 ± 1.36 ^d^	7.1 ± 0.29 ^c^

All statistical differences were submitted regarding the control variant. The data are averages from three SE replications; the averages with different letters are significantly different (*p* ≤ 0.05).

**Table 2:** Influence of 200 µM SNP on the content of GSH, GSSG (μmoL mg^−1^ protein), GSH/GSSG ratio, ASA (mg% FW), MDA (nM g^−1^ FW), H₂O₂, proline (µmoL g^−1^ FW), and antioxidant enzymes GR (nmoL min^−1^ mg^−1^ protein) and APX (µmoL ascorbate oxidized mg^−1^ min^−1^ protein) in leaves of wheat seedlings under salinity stress. Time of stress exposure (100 mM NaCl)–60 days.

Treatment	GSH	GSSG	GSH/ GSSG	ASA	GR	APX	H_2_O_2_	Proline	MDA
Control	90 ± 3.6 ^b^	8.9 ± 0.36 ^a^	10.1 ± 0.4 ^b^	2.85 ± 0.11 ^a^	5.35 ± 0.21 ^a^	8.17 ± 0.32 ^a^	5.25 ± 0.21 ^a^	20.4 ± 0.82 ^a^	8.25 ± 0.33 ^a^
200µM SNP	93.9 ± 3.6 ^b^	9.1 ± 0.37 ^a^	10.3 ± 1.41 ^b^	3 ± 0.12 ^a^	6.2 ± 0.25 ^b^	8.6 ± 0.34 ^b^	5.89 ± 0.23 ^b^	21.3 ± 0.85 ^ab^	8.34 ± 0.33 ^a^
NaCl	30 ± 1.2 ^a^	12.5 ± 0.5 ^b^	2.4 ± 0.09 ^a^	1 ± 0.04 ^b^	9.3 ± 0.37 ^c^	15 ± 0.61 ^c^	9.1 ± 0.37 ^c^	35.8 ± 1.4 ^c^	16.2 ± 0.64 ^b^
(SNP) + NaCl	54 ± 2.2 ^c^	9 ± 0.36 ^a^	6 ± 0.24 ^c^	2.1 ± 0.08 ^c^	9.8 ± 0.39 ^d^	9.8 ± 0.39 ^d^	7.2 ± 0.29 ^d^	37.8 ± 1.5 ^d^	9.8 ± 0.39 ^c^

All statistical differences were submitted regarding the control variant. The data are averages from three SE replications; the averages with different letters are significantly different (*p* ≤ 0.05).

**Table 3:** Effect of SNP pretreatment on yield components of wheat plants. The table presented contains average data for the years 2021 and 2022.

Year	Treatments	Shoot Length (cm)	Spike Length (cm)	1000 Grain Weight (g)
2021	Control	101 ± 4.1 ᵃ	9.3 ± 0.37 ᵃ	36.8 ± 1.48 ᵃ
	200 µM SNP	114 ± 4.6 ᵇ	12 ± 0.48 ᵇ	44.2 ± 1.76 ᵇ
2022	Control	109 ± 4.4 ᵃ	10.4 ± 0.4 ᵃ	39.3 ± 1.57 ᵃ
	200 µM SNP	123 ± 4.9 ᵇ	13 ± 0.52 ᵇ	47.1 ± 1.88 ᵇ

All statistical differences were submitted regarding the control variant. The data are averages from three SE replications; the averages with different letters are significantly different (*p* ≤ 0.05).

**Table 4:** Effect of SNP pretreatment on amino acid (AA) profiles, content of carbohydrate (mg 100 g^−1^ DW), and raw fat (%) of wheat grains.

Parameters	Control	200 µM SNP
Valine	408.07 ± 16.32 ^a^	511.75 ± 20.50 ^b^
Leucine + Isoleucine	995.42 ± 39.80 ^a^	1140.39 ± 45.60 ^b^
Methionine	151.42 ± 6.04 ^a^	233.48 ± 9.36 ^b^
Tryptophan	180.81 ± 7.20 ^a^	255.61 ± 10.2 ^b^
Phenylalanine	426.43 ± 17.04 ^a^	556.31 ± 22.24 ^b^
Lysine	327.39 ± 13.08 ^a^	455.09 ± 18.2 ^b^
Threonine	315.10 ± 12.6	421.09 ± 16.84 ^b^
Histidine	289.92 ± 11.55 ^a^	391.89 ± 15.67 ^b^
Essential AA	3091 ± 123.65 ^a^	3962 ± 158.42 ^b^
Arginine	610.67 ± 24.4 ^a^	800.21 ± 32.90 ^b^
Serine	557.33 ± 22.28 ^a^	700.28 ± 28.65 ^b^
Proline	1054.35 ± 42.15 ^a^	1270.28 ± 50.8 ^b^
Glycine	419.93 ± 16.74 ^a^	500.76 ± 20.86 ^b^
Alanine	357.47 ± 14.67 ^a^	420.49 ± 16.8 ^b^
Aspartic acid + Asparagine	401.72 ± 16.01 ^a^	422.53 ± 16.91 ^a^
Glutamic acid	3101.82 ± 124.04 ^a^	3275.39 ± 131.22 ^a^
Cysteine	262.26 ± 10.60 ^a^	261.45 ± 10.17 ^a^
Non-essential AA	6761 ± 270.45 ^a^	7693 ± 307.75 ^b^
Total AA	9852 ± 392.26 ^a^	11655 ± 466.65 ^b^
Essential AA/Non-essential AA	0.45 ± 0.02 ^a^	0.52 ± 0.03 ^b^
Fructose	108.67 ± 4.32 ^a^	116.93 ± 4.62 ^a^
Glucose	271.23 ± 10.84 ^a^	278.38 ± 11.19 ^a^
Sucrose	1204.09 ± 48.15 ^a^	1248.32 ± 49.28 ^a^
Raw fat	2.54 ± 0.10 ^a^	2.57 ± 0.10 ^a^

All statistical differences were submitted regarding the control variant. The data are averages from three SE replications; the averages with different letters are significantly different (*p* ≤ 0.05).

## Data Availability

Not applicable.
